# Involvement of the Akt/NF-κB Pathways in the HTNV-Mediated Increase of IL-6, CCL5, ICAM-1, and VCAM-1 in HUVECs

**DOI:** 10.1371/journal.pone.0093810

**Published:** 2014-04-08

**Authors:** Haitao Yu, Wei Jiang, Hong Du, Yuan Xing, Guangzhen Bai, Ye Zhang, Yu Li, Hong Jiang, Ying Zhang, Jiuping Wang, Pingzhong Wang, Xuefan Bai

**Affiliations:** 1 Department of Infectious Diseases, Tangdu Hospital, Fourth Military Medical University, Xi’an, Shaanxi Province, China; 2 Department of Physiology, Fourth Military Medical University, Xi’an, Shaanxi Province, China; 3 Department of Thoracic Surgery, Tangdu Hospital, Fourth Military Medical University, Xi’an, Shaanxi Province, China; 4 Department of Infectious Diseases, Shaanxi Provincial People's Hospital,Xi’an, Shaanxi Province, China; University of Alabama at Birmingham, United States of America

## Abstract

**Background:**

Hantaan virus (HTNV) infection causes a severe form of HFRS(hemorrhagic fever with renal syndrome)in Asia. Although HTNV has been isolated for nearly forty years, the pathogenesis of HFRS is still unknown, and little is known regarding the signaling pathway that is activated by the virus.

**Methodology/Principal Findings:**

Cardamonin was selected as a NF-κB inhibitor, and indirect immunofluorescence assays were used to detect the effect of cardamonin on HTNV-infected HUVECs. The effect of cardamonin on the HTNV-induced phosphorylation of Akt and DNA-binding activity of NF-κB were determined using Western blot analysis and electrophoretic mobility shift assays (EMSAs), respectively. Then, flow cytometric and quantitative real-time PCR analyses were performed to quantify the expression levels of the adhesion molecules ICAM-1 and VCAM-1, and the concentrations of IL-6, IL-8, and CCL5 in HUVEC supernatants were examined using ELISA. The results showed that cardamonin did not effect the proliferation of HUVECs or the replication of HTNV in HUVECs. Instead, cardamonin inhibited the phosphorylation of Akt and nuclear transduction of NF-κB and further reduced the expression of the adhesion molecules ICAM-1 and VCAM-1 in HTNV-infected HUVECs. Cardamonin also inhibited the secretion of IL-6 and CCL5, but not IL-8.

**Conclusion/Significance:**

HTNV replication may not be dependent upon the ability of the virus to activate NF-κB in HUVECs. The Akt/NF-κB pathways may be involved in the pathogenesis of HFRS; therefore, cardamonin may serve as a potential beneficial agent for HFRS therapy.

## Introduction

Hantaviruses are enveloped, negative-sense RNA viruses from the genus *Hantavirus* within the family *Bunyaviridae* [1]. These reservoir animals are asymptomatic following infection, although persistent infection is established and high titers of neutralizing antibodies accumulate [2]. Therefore, rodents, the main kinds of reservoir animals, serve as natural viral reservoirs. Unlike in rodents, hantavirus infection leads to two different forms of severe febrile disease in humans, hemorrhagic fever with renal syndrome (HFRS) [3] and hantavirus pulmonary syndrome (HPS) [4]. Approximately 100,000 cases of HFRS are documented annually, with a mortality rate of 2%–10% [5]. More than 90% of these cases occur in Asian countries, including China, Russia, and Korea.

Hantaan virus (HTNV), a species of the genus *Hantavirus*, causes a severe form of HFRS. It is a noncytopathogenic virus and, consequently, is characterized by increased vascular permeability and coagulation disorders [6]. Therefore, the pathogenesis of HFRS is not associated with a direct virus effect. Most likely, this disease develops via indirect mechanisms including the immune response, up or down regulation of cytokines and chemokines, immune complexes (ICs), and complement activation. Tumor necrosis factor (TNF), interleukin (IL-6 and IL-10), vascular endothelial growth factor, and cytotoxic T cell-mediated mechanisms are more likely responsible for the symptoms observed in HFRS [7], [8], [9], [10]. Increased levels of TNF, IL-1, IL-6, IL-8, IFN-γ, IP-10, and CCL5 were observed *in vivo* and *in vitro* [11], [12], [13], and several cytokines, such as IFN-γ, IL-1α, IL-6, and TNF, were also detected in lung tissues of HPS cases [14]. Intercellular adhesion molecule type 1 (ICAM-1) and vascular cell adhesion molecule type 1 (VCAM-1) provide costimulatory signals that activate T lymphocytes. These cell adhesion molecules, which are expressed on the surface of endothelial cells, play an essential role in leukocyte adherence and propagation of inflammatory responses [15]. The expression of sICAM-1 and sVCAM-1 was up-regulated in HFRS patients and correlated with the pathogenesis of HFRS [16], [17].

NF-κB represents a family of eukaryotic transcription factors involved in the early pathogen response, which plays an important role in promoting inflammation and viral gene expression [18], [19]. NF-κB normally exists as an inactive cytoplasmic complex that is bound to inhibitory proteins of the IκB family [20]. Release of IκBs results in nuclear translocation of NF-κB and its binding to DNA at specific κB sites, which rapidly induces a variety of genes encoding, among others, cell adhesion molecules, cytokines, chemokines, cytokine receptors, and enzymes that produce inflammatory mediators [21]. It is well known that different DNA and RNA virus infections can trigger NF-κB activation via different signaling mechanisms [20]. Akt, a kinase upstream of IKK, seems to enhance nuclear translocation of NF-κB through phosphorylation and activation of IκB kinase, resulting in enhanced degradation of IκBα [22], [23]. However, little is known about the signaling pathway that is activated by the virus and the role of the nuclear factor in the infection process during HTNV infection.

To elucidate the role of NF-κB in HTNV-infected Human Umbilical Vein Endothelial Cells (HUVECs), cardamonin (2′, 4′-dihydroxy-6′-methoxychalcone), which was isolated from the spicy and herbaceous plant *Alpinia conchigera* Griff (Zingiberaceae), was chosen as an inhibitor of NF-κB activation [24], [25]. The expression levels of several cytokines (TNF, IL-6, IL-1β), chemokines (CCL5, IL-8), and adhesion molecules (ICAM-1, VCAM-1) were detected in the supernatants of HUVECs infected with HTNV. Then, the capacity of cardamonin to inhibit the nuclear translocation of NF-κB and the phosphorylation of Akt were analyzed. For the first time, we demonstrate that the Akt/NF-κB pathways may be involved in the expression of these molecules in HTNV-infected HUVECs, and cardamonin may serve as a potential, beneficial agent for HFRS therapy.

## Materials and Methods

### Cells and virus

Human umbilical vein endothelial cells (HUVECs) were isolated from human umbilical cords as previously described [26] and cultured in Endothelial Cell Medium (San Diego, CA), containing essential and non-essential amino acids, vitamins, organic and inorganic compounds, hormones, growth factors, trace minerals, and a low concentration of fetal bovine serum (FBS) (5%) at 37°C in humidified air containing 5% CO_2_. The cells were used at fewer than 8 passages in this study.

HTNV, strain 76–118 [27], was proliferated in Vero E6 cells and titered using an immunofluorescence staining assay for HTNV nucleocapsid protein as previously described [28]. The TCID_50_ was 10^5.5^/ml and calculated using the Reed-Muench method.

During all experiments, the cells were pretreated with 30 μM cardamonin (Tocris Bioscience, Bristol, UK) for 30 min [25] and then incubated with or without HTNV for different times.

### Indirect immunofluorescence assays

Indirect immunofluorescence assays (IFA) were performed as previously described [29]. HUVECs were cultured on glass coverslips until semi-confluence, pretreated with cardamonin for 30 min, and then incubated with HTNV. At 24 h and 72 h post HTNV infection, the coverslips were developed for IFA. Following incubation with mouse monoclonal antibody against HTNV (prepared by the Department of Microbiology, The Fourth Military Medical University, Xi’an, Shaanxi, China; dilution 1∶1000) for 2 h at 37°C, the cells were washed three times with PBS for 5 min per wash. Then, the cells were incubated with fluorescein isothiocyanate (FITC)-conjugated goat anti-mouse IgG (SouthernBiotech, Birmingham, Alabama, USA; dilution 1∶500) for 1 h at 37°C. Last, the nuclei were stained with 0.01% Evans blue, and an Olympus BX51 fluorescence microscope (Olympus, Tokyo, Japan) with the appropriate fluorescence filters was used to capture the images.

### Western blot analysis

Cell extracts were prepared as previously described [30]. Subsequently, 10 μg of total protein from each sample was added to Laemmli loading buffer, boiled for 5 min, resolved using 10% SDS-PAGE, and electroblotted onto polyvinylidene difluoride (PVDF) membrane (Millipore, Billerica, MA). After blocking with 3% BSA at room temperature for 30-60 min, the membrane was respectively incubated with antibodies against Akt or p-Akt (Cell Signaling Technology, Boston, Massachusetts) overnight at 4°C. The membrane was then washed with PBST and incubated with horseradish peroxidase-conjugated IgG antibody (Cell Signaling Technology, Boston, Massachusetts) for 1 h at room temperature. The blots were developed using an enhanced chemiluminescence detection kit (Millipore, Billerica, MA), the immunoblotting was visualized using a ChemiDoc XRS (Bio-Rad Laboratory, Hercules, CA), and the blot densities were analyzed using the Quantity One software.

### Electrophoretic mobility shift assay (EMSA)

Nuclear extracts from HUVECs were prepared using a nuclear protein extraction kit (Viagene Biotech, Ningbo, China). EMSAs were performed using a non-radioactive EMSA kit in accordance with the manufacturer's instructions (Pierce, Rockford, IL). The sequence of the oligonucleotide, which was biotinylated at its 5′ end, was as follows: 5′-AGTTGAGGGGACTTTCCCAGGC-3′. Crude nuclear protein samples (10 μg) were incubated for 20 min at room temperature in a binding reaction mixture containing 1.5 μL 10× binding buffer, 1.5 μL poly(dI-dC) (1.0 μg/μL), and ddH_2_O to a final volume of 14.4 μL. Next, 0.6 μL (300 fmol) of the probe was added, and the reaction was incubated for 20 min at room temperature. Where indicated, 2 μL of specific, cold-competitor oligonucleotides in 100× competing buffer was added before the labeled probe, and the reaction was incubated for 20 min. Protein-DNA complexes were resolved using electrophoresis at 4°C in a 6.5% acrylamide gel and subjected to chemiluminescence. Electrophoresis was performed using a 6.5% non-denaturing polyacrylamide gel at 175 V in 0.25×TBE (1×TBE, 89 mM Tris-HCl, 89 mM boric acid, and 5 mM EDTA, pH 8.0) at 4°C for 1 h. The gels were transferred to the bonding membrane at 394 mA in 0.5×TBE at room temperature for 40 min. After cross-linking for 10 min with a UV cross-linking apparatus (immobilization), the membrane was blocked, streptavidin-HRP labeled, washed again, and equilibrated; images were captured using an Imager apparatus (Alpha Innotech, San Leandro, CA).

### Flow cytometric analysis of ICAM-1 and VCAM-1 expression

HUVECs were cultured in 6-well plates. Upon confluency, the cells were treated as previously described. HUVECs were incubated for 48 h with or without HTNV. After stimulation, the cells were washed with PBS and treated for 30 min at 4°C in the dark with saturating amounts of FITC-conjugated anti–ICAM-1 (Biolegend, San Diego, CA) and APC-conjugated anti–VCAM-1 (Biolegend, San Diego, CA) monoclonal antibodies. Cells were then washed with PBS, fixed in 4% paraformaldehyde, harvested by mild trypsinization, washed again, and centrifuged at 300 g for 5 min. Then, the cells were resuspended and analyzed using flow cytometry (FACSCalibur, Becton Dickinson, Franklin Lakes, NJ). The data were analyzed using FlowJo version 7.6.2 for Windows (Tree Star Inc., Ashland, OR).

### Quantitative real-time PCR analysis of ICAM-1 and VCAM-1 expression

Total RNA was extracted, and cDNA was synthesized, as described above [31]. Quantitative real-time PCR was performed using the SYBR Premix Ex Taq II (Takara Biotechnology Co., Dalian, China). The levels of each RNA were normalized to that of the housekeeping gene GAPDH. The sequences of the oligonucleotides (synthesized by Takara Biotechnology Co., Dalian, China) were as follows: ICAM-1 (forward, 5′-AGCCAACCAATGTGCTATTCAAAC-3′ and reverse, 5′-CACCTGGCAGCGTAGGGTAA-3′); VCAM-1 (forward, 5′-CGAAAGGCCCAGTTGAAGGA-3′ and reverse, 5′-GAGCACGAGAAGCTCAGGAGAAA-3′); and GAPDH (forward, 5′-GCACCGTCAAGGCTGAGAAC-3′ and reverse, 5′-TGGTGAAGACGCCAGTGGA-3′). The results were analyzed according to the manufacturer's protocol.

### Cytokine/Chemokine measurement

Culture supernatants were harvested and preserved at −80°C before evaluation of cytokine production using ELISA. The concentrations of TNF, IL-1β, IL-6, IL-8, and CCL5 were measured using a commercial enzyme linked immunosorbent assay (ELISA) kit (Bender MedSystems GmbH, Vienna, Austria) according to the manufacturer's instruction.

### Statistical analysis

All data are expressed as the mean ± SD of three independent experiments with each experiment performed in triplicate. The statistical significance was calculated using Student's t-test, and a significant value of *P*<0.05 was considered to be statistically significant.

## Results

### 1. Equal infection efficiency of HTNV with or without cardamonin treatment

**Figure 1:**
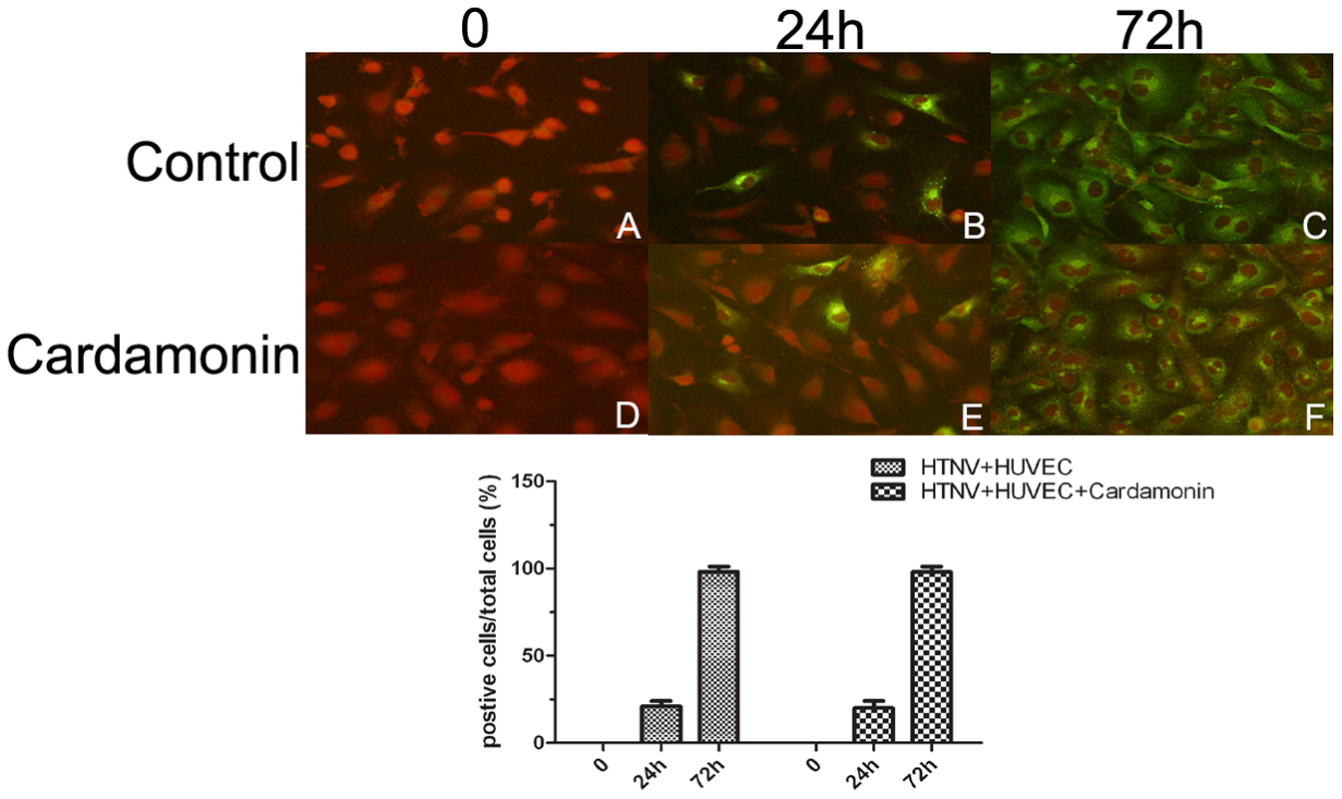
Detection of HTNV infection efficiency in HUVECs with or without cardamonin treatment (CD). The upper panel: A, normal HUVECs without any treatment; B, HUVECs infected with HTNV for 24 hours; C, HUVECs infected with HTNV for 72 hours. D, E, F, HUVECs pretreated with 30 μM cardamonin for 30 min before HTNV infection; D, cardamonin-pretreated HUVECs; E, cardamonin-pretreated HUVECs infected with HTNV for 24 hours; F, cardamonin-pretreated HUVECs infected with HTNV for 72 hours. Original magnifications, ×200. Lower panel: data are shown as the ratio of positive cells/total cells from five independent microscopic fields.

To determine whether the use of cardamonin interfered with the proliferation of HUVECs and the replication of HTNV in HUVECs, IFA was used to determine the percentage of infected cells in total cells. The results showed that approximately 20% of the total cells were infected with HTNV at 24 h (Fig. 1.B), and nearly all of the cells were infected with HTNV at 72 h (Fig. 1.C). Similar results were obtained in the cardamonin-pretreated groups (Fig. 1.E.F). As shown in Fig. 1.A.D, the negative control groups did not show any green fluorescence. The lower panel shows no significant difference between virus-positive and total cells or normal and cardamonin-pretreated HUVECs at 24 h or 72 h after HTNV infection. The results also indicated no significant differences in the cell densities of the relative groups, and cell viability was not affected by cardamonin treatment.

### 2. Cardamonin inhibits Akt Activation in HTNV-infected HUVECs

**Figure 2:**
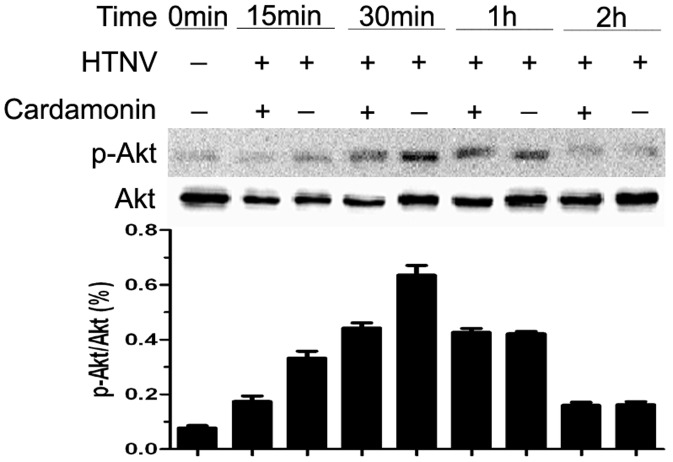
Effect of cardamonin on the HTNV-induced activation of Akt. The upper panel, HUVECs were pretreated for 30-Akt was detected by Western blot analysis as described before. The bottom panel represents Akt to show equal loading of cell lysates. Data are shown as the ratio of the gray scale for p-Akt and Akt.

To further investigate how cardamonin prevents NF-κB activation, we examined the effect of cardamonin on the HTNV-induced activation of Akt. HUVECs were pretreated with cardamonin for 30 min and then infected with HTNV for the indicated time points (Fig. 2). Infection of HUVECs with HTNV significantly induced the phosphorylation of Akt, with maximum phosphorylation occurring at 30 min. In contrast, pretreatment with cardamonin suppressed HTNV-induced phosphorylation of Akt.

### 3. Cardamonin inhibits nuclear translocation of NF-κB by HTNV

**Figure 3:**
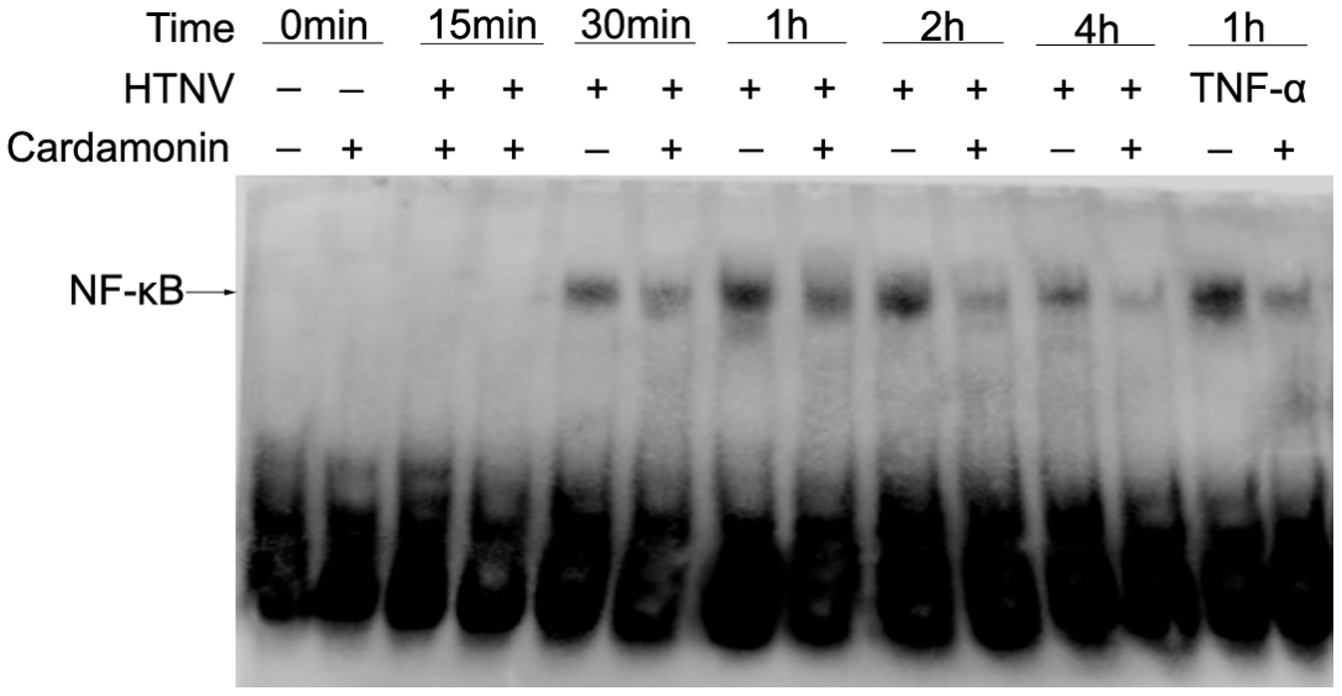
Effect of cardamonin on the HTNV-induced nuclear translocation of NF-κB. HUVECs were preincubated for 30-κB using EMSA as described before. A representative autoradiographic exposure is shown, and the arrow indicates the location of the DNA-NF-κB complexes. TNF-α-stimulated cells were used as positive control groups.

As shown in Fig. 3, the activation of NF-κB in HUVECs following HTNV infection was detectable as early as 30 min after infection, and pretreatment with cardamonin prevented this activation. The peak point occurred at 2 h and demonstrated that inhibition of Akt phosphorylation was effective in blocking the nuclear transduction of NF-κB that was elicited by HTNV infection in HUVECs. TNF-α-stimulated cells were selected as positive groups and obtained similar results.

### 4. Cardamonin inhibits the expression of ICAM-1 and VCAM-1

**Figure 4:**
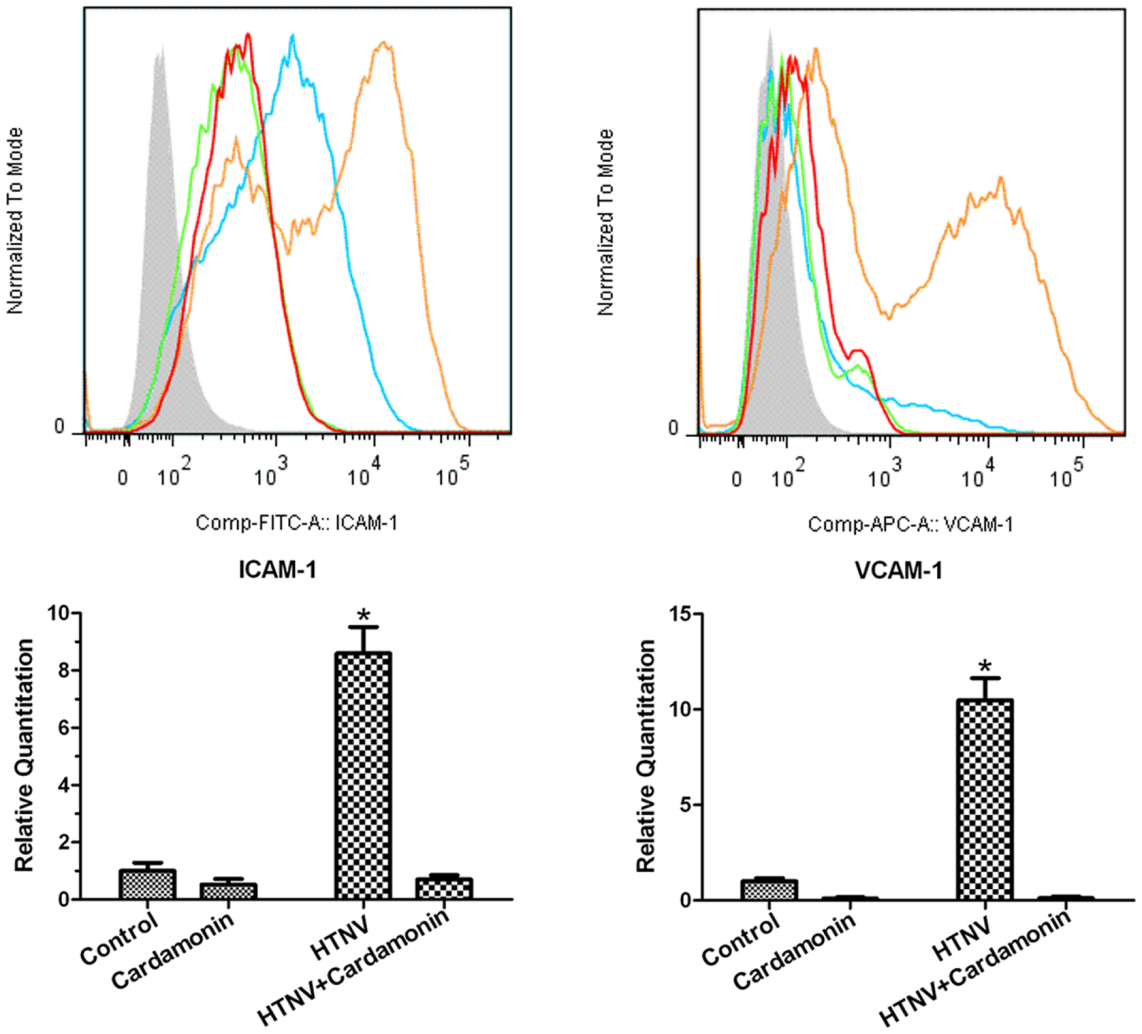
Detection of the expression of ICAM-1 and VCAM-1 in HTNV-infected HUVECs with or without cardamonin treatment. HUVECs were pretreated for 30 μM cardamonin followed by infection with HTNV for the indicated time points. Subsequently, flow cytometric analysis and quantitative real-time PCR analysis were performed as described before. HTNV induced the expression of ICAM-1 and VCAM-1 at the mRNA and protein levels, and this phenomenon was effectively inhibited by cardamonin (*, *p*<0.05).

The VCAM-1 and ICAM-1 expression levels on the surface of HUVECs were analyzed using flow cytometry and quantitative real-time PCR. The results showed that HUVECs cultured with HTNV demonstrated markedly enhanced VCAM-1 and ICAM-1 expression at the mRNA and protein levels compared with resting cells (Fig. 4). However, cells pretreated with cardamonin showed significantly reduced VCAM-1 and ICAM-1 expression, and these levels were even lower than those in LPS-stimulated HUVECs (data not shown).

### 5. Cardamonin inhibits the secretion of IL-6 and CCL5, but not IL-8, in HTNV-infected HUVECs

**Figure 5:**
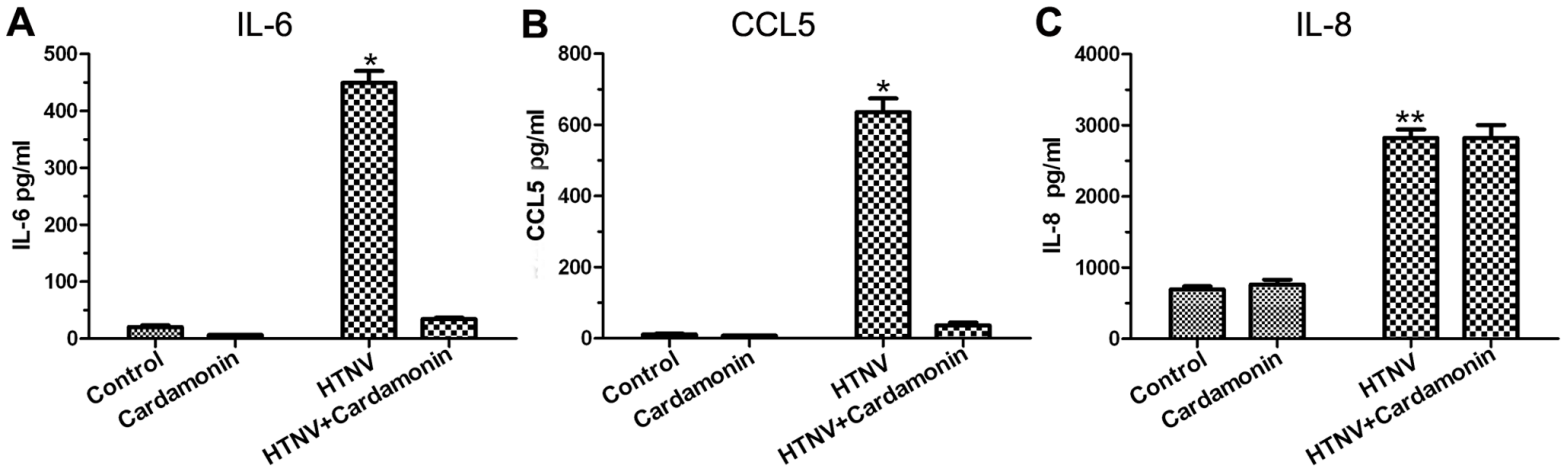
Detection of the expression of IL-6, CCL5, and IL-8 in HTNV-infected HUVECs with or without cardamonin treatment. HUVECs were pretreated with 30 μM cardamonin for 30 min followed by infection with HTNV for 48 h. Subsequently, ELISA was performed as described before. HTNV induced the expression of IL-6, IL-8, and CCL5 in HUVECs. However, cardamonin effectively inhibited the secretion of IL-6 and CCL5 (A, B), but not IL-8 (C), in HTNV-infected HUVECs. (*, *p*<0.05 compared with all other groups; **, *p*<0.05 compared with the control group, *p*>0.05 compared with the HTNV + cardamonin group).

To determine whether HTNV infection-triggered inflammatory responses are caused by activation of the Akt/NF-κB pathways, we examined various cytokines and chemokines that are known to act downstream of NF-κB. The increased secretion of IL-6, IL-8, and CCL5 were examined using ELISA in the supernatants of HTNV-infected and resting HUVECs (Fig. 5). We found that cardamonin only suppressed the secretion of IL-6 and CCL5, while no significant difference in the expression level of IL-8 was detected. The levels of TNF and IL-1β in HTNV-infected HUVECs and uninfected cells were not detectable (data not shown).

## Discussion

Viruses have long been known to utilize a variety of strategies to penetrate host cells. Recently, studies on the elaborate relationship between viruses and their hosts has led to an understanding of how viral pathogens are able to alter the host metabolism via their signaling proteins and are able to hijack cellular signaling pathways and transcription factors and control them to their own advantage. NF-κB is a critical regulator of the immediate early pathogen response and plays an important role in promoting inflammation and in the regulation of cell proliferation and survival [18], [19]. These responses involve a plethora of cytokines and chemokines, receptors required for neutrophil adhesion and transmigration across blood vessel walls, as well as proteins involved in antigen presentation [32], which may be responsible for the pathogenesis of some virus-induced diseases. Many viruses, including several human pathogens such as HIV-1, the human T-cell leukemia virus-1 (HTLV-1), influenza virus, hepatitis B and C viruses, as well as herpeviruses, have evolved different strategies to modulate the NF-κB pathway [20], [33].

NF-κB activation during viral infection has been interpreted in some cases as a protective response by the host to viral pathogens, whereas, in other cases, it has been shown that the virus utilizes the factor to enhance its replication [20]. Several viral pathogens, including HIV and herpes simplex viruses (HSV), were reported to activate NF-κB to enhance viral transcription and replication [34], [35]. It is said that infection by these viruses triggers the activation of NF-κB and then activates its κB-containing viral promoter to enhance viral transcription. In contrast, several studies, such as those in HCMV or influenza A virus-infected cells, showed contrary evidence. These results indicated that viral replication is independent of the ability of the viruses to activate NF-κB, and activation of the NF-κB pathway is dispensable for virus replication [33], [36], [37], [38]. In the case of influenza A virus-infected cells, the H1N1 virus induced NF-κB activation in A549 cells and caused a productive infection, with high levels of viral particles that were produced at 24 h post infection. However, H1N1 virus infection was unable to induce NF-κB to 24 h post infection in human kidney 293 cells, which are equally permissive to the virus. In addition, H1N1 virus did not induce NF-κB activation in two other human non-pulmonary cell lines that are susceptible to viral infection. Although no activation of NF-κB could be detected in MDCK cells, the H1N1 virus yield was higher than that in any of the other tested cell lines [33]. In the present study, we detected the ratio of fluorescence-positive cells, which indirectly represent virus replication, in total HUVECs at 24 h and 72 h post infection by HTNV. Statistical analysis showed that no significant difference in the fluorescence-positive/total cells ratios of the cardamonin treated and non-treated groups was detected. The following results also showed that HTNV induced NF-κB activation immediately after infection and that cardamonin inhibited the activation of NF-κB. Preliminary experiments indicated that HTNV replication is independent upon the ability of the virus to activate NF-κB in HUVECs.

NF-κB activation in response to external pro-inflammatory stimuli, such as mitogens, cytokines, and lipopolysaccharide (LPS), is involved in the rapid inhibition of κBα (I-κBα) phosphorylation by I-κB kinase (IKK). Akt, a downstream target for activated phosphatidylinositol 3-kinase (PI 3-kinase) [39], [40], is activated by mitogens and cytokines that act as survival factors. It mediates its functions by phosphorylating substrates that decrease the activity of pro-apoptotic proteins or increase the activity of anti-apoptotic proteins [39], [41], [42]. Furthermore, Akt can activate a member of the mitogen-activated protein kinase kinase kinase (MAP3K) family, Cot, and indirectly affect the activity of IKK and NF-κB [43]. Thus, PI3-kinase/Akt signaling occurs upstream of diverse pathways that activate NF-κB, and the roles of the Akt/NF-κB pathways in many diseases have been discussed in recent years [44], [45], [46], [47], [48]. However, the mechanisms through which PI3-kinase/Akt signaling activates NF-κB are cell type-specific. A previous study showed that a inhibitor of PI3-kinases, wortmannin, completely blocked TNF-induced NF-κB DNA binding in 293 cells, and partially blocked binding in MCF-7, ME-180, Hela, and NIH 3T3 cells, but had little effect on binding in H1299 cells and HUVECs. Although TNF activated Akt in each cell type [49], Balwani S, *et al*. demonstrated that a novel compound, 2-methyl-pyran-4-one-3-O-β-D-2′,3′,4′,6′-tetra-O-acetyl glucopyranoside (MPTAG), could inhibit the activation of NF-κB through a PI3K-independent and PKA/Akt-dependent pathway in TNF-stimulated HUVECs [47]. In the present study, we also revealed that HTNV induced the activation of Akt at 15 min and then induced the activation of NF-κB at 30 min. Akt/NF-κB pathways may be involved in HTNV-infected HUVECs, and the different results of previous studies may be due to different signaling pathways of TNF-stimulated cells and HTNV-infected cells or the different inhibitors that were chosen in the two experiments. However, small molecules that act as inhibitors of different pathways may be non-specific, e.g., the mechanism of cardamonin in HTNV-infected HUVECs, and require further discussion.

Human vascular endothelial cells are thought to be the primary sites of HTNV replication which results in infected and/or adjacent cells expressing high levels of cytokines/chemokines and adhesion molecules [10], [50], [51], [52]. Increased capillary permeability is considered to be a common, underlying factor of HFRS [6], [53]. Because HTNV is a noncytopathogenic virus, the pathogenesis of HFRS is not associated with a direct virus effect. Therefore, it is assumed that capillary leakage is more likely to be caused by redundant cytokines/chemokines and adhesion molecules. Significantly elevated plasma levels of IL-6, IL-8, and CCL5 were detected at the onset of the acute phase of HFRS [12], [54], and higher concentrations were observed in more severe disease types [12]. Increased expression of IL-6 is associated with clinically severe Nephropathia epidemica (NE), and high IL-6 was found to be an independent risk factor for impaired renal function, thrombocytopenia, and longer hospitalization [55]. In the present study, we examined the levels of TNF, IL-1β, IL-6, IL-8, and CCL5 in the supernatants of HTNV-infected HUVECs at 48 h post infection using ELISA. Increased IL-6, IL-8, and CCL5 were detected in HTNV-infected cells when compared with uninfected cells. However, no detectable levels of TNF or IL-1β in HTNV-infected HUVECs or uninfected cells were observed. In contrast to our findings, Jiang et al. reported high levels of TNF and IL-6 in HTNV-infected EVC-304 cells, but no significant difference in the expression of IL-8 before and after HTNV infection was observed [56]. Sundstrom et al. also reported that no virus-specific cytokines/chemokines, including TNF, IL-6, IL-1β, IL-8, were detected at either the protein or message level in hantavirus-infected human lung microvascular endothelial cells (HMVEC-Ls), but the expression of CCL5, which is predominantly chemotactic for mononuclear leukocytes, was enhanced by hantavirus infection [13]. One difference between our experiment and that of others was the cells that were utilized. We used HUVECs, while the others used EVC-304 cells or HMVEC-Ls. In cardamonin-pretreated cells, the increased expression of IL-6 and CCL5 was suppressed via the Akt/NF-κB pathways. However, cardamonin treatment did not influence the expression of IL-8. In contrast to our results, Shah A. et al reported that the Akt/NF-κB pathways were involved in the methamphetamine-mediated increase in IL-6 and IL-8 expression in astrocytes [45]. The same group also reported another case where HIV gp120 and methamphetamine affected IL-6, but not IL-8, via the Akt/NF-κB pathways [57]. The latter report is in agreement with our results, and it is possible that other signaling pathways, rather than Akt/NF-κB, could regulate the secretion of IL-8 from HTNV-infected HUVECs. In our study, we also detected the expression of ICAM-1 and VCAM-1, which provide costimulatory signals for the activation of T lymphocytes. These cell adhesion molecules, which are expressed on the endothelial cell surface, play an essential role in leukocyte adherence and propagation of inflammatory responses [15], [58]. Increased levels of ICAM-1 and VCAM-1 were observed in HTNV-infected HUVECs, and further research indicated that the expression of ICAM-1 and VCAM-1 were regulated by the Akt/NF-κB pathways. These observations can be used to describe a possible scenario of increased vascular permeability in HFRS.

The study preliminarily elucidates that HTNV replication is independent of the ability of the virus to activate NF-κB in HUVECs. The selective induction of cytokines/chemokines and adhesion molecules may provide a clue into the involvement of the specific immune response in the pathogenesis of HFRS, and blockade of the Akt/NF-κB pathways could reduce the excessive inflammatory reaction. The Akt/NF-κB pathways could be involved in the expression of these molecules in HTNV-infected HUVECs; therefore, cardamonin could be a potential beneficial agent for HFRS therapy.
